# Target-Guided Query Allocation and Boundary-Aware Multi-Scale Feature Fusion for Object Detection in UAV Imagery

**DOI:** 10.3390/s26196101

**Published:** 2026-09-26

**Authors:** Na Chen, Caichen Han, Zhongyuan Jiang, Shuo Liu, Baozhou Chen

**Affiliations:** School of Mathematics and Computer Science, Wuhan Polytechnic University, Wuhan 430048, China; 15374556951@163.com (N.C.); 18768990202@163.com (C.H.); 13024008015@163.com (Z.J.); ls201108@whpu.edu.cn (S.L.)

**Keywords:** UAV remote sensing, small-object detection, TQ-DETR, dynamic queries, objectness modeling, feature fusion

## Abstract

Object detection in unmanned aerial vehicle (UAV)-based visual sensing is important for aerial monitoring and intelligent perception. However, UAV imagery often contains small and densely distributed objects, complex backgrounds, and substantial scale variations. Repeated downsampling weakens the boundary and localization cues of small objects, while query initialization based mainly on classification responses may overlook objects with weak semantic features. To address these challenges, we propose TQ-DETR, a target-guided end-to-end detector based on RT-DETRv2. A Target-Oriented Branch (TOB) learns class-agnostic objectness priors to provide reliable foreground spatial guidance independent of category confidence. Based on this prior, Dynamic Query Allocation (DQ) supplements semantic queries with objectness-guided candidates under a fixed query budget, improving candidate coverage for small objects with weak classification responses. In addition, Objectness-Guided Multiscale Boundary Fusion (OG-MBF) selectively extracts and injects target-relevant shallow boundary information while suppressing high-frequency background interference, thereby recovering fine-grained localization cues without adding an extra Transformer feature level. Experiments on VisDrone2019 show that, over three independent runs, TQ-DETR achieved an AP of 30.21 ± 0.51% and an AP50 of 50.28 ± 0.65%, outperforming RT-DETRv2-R18 by 1.79% and 2.75%, respectively, demonstrating improved detection of small and densely distributed UAV targets.

## 1. Introduction

Object detection in UAV remote sensing imagery supports applications such as traffic monitoring, urban management, public safety inspection, and disaster response. Compared with conventional ground-view imagery, UAV imagery has a bird’s-eye perspective, a large field of view, complex backgrounds, dense object distributions, and substantial scale variation. Repeated downsampling can degrade the texture, boundary, and semantic information of small objects, while dense distributions increase competition among neighboring instances. UAV detectors must therefore preserve fine-grained representations and maintain adequate candidate coverage under constrained computational budgets.

VisDrone2019 [1] provides a representative benchmark for evaluating UAV object detection under these conditions. It contains ten object categories and includes scenes with frequent occlusion, large scale variation, complex backgrounds, and uneven spatial distributions. Roads, intersections, and parking areas often contain dense object clusters, whereas other image regions are dominated by background. This spatial imbalance reduces the efficiency of uniform full-image computation and increases the risk that candidate selection based only on classification confidence will omit valid objects with weak responses.

To improve the efficiency of UAV object detection in high-resolution aerial imagery, numerous studies have introduced spatial priors to guide computational resource allocation. ClusDet performs local detection by identifying target-dense regions [2], ESOD reduces redundant computation through target-oriented region search and feature-level slicing [3], and QueryDet predicts query locations from low-resolution features and performs sparse detection on high-resolution feature maps [4]. To improve localization accuracy for small objects, NWD models bounding boxes using Gaussian distributions and measures their similarity with the normalized Wasserstein distance [5]. Collectively, these studies show that spatial priors can guide detectors toward regions with a higher probability of containing objects. However, they address region selection, sparse computation, or localization-oriented label assignment rather than jointly improving shallow-detail preservation and decoder candidate coverage.

To improve aerial-object representations, numerous multiscale fusion strategies have been proposed. FPN constructs hierarchical semantic representations through top-down pathways and lateral connections [6], while EFPN exploits high-resolution features and texture propagation [7]. TPH-YOLOv5 adds a Transformer prediction head and an additional detection scale [8], Drone-YOLO integrates shallow features with multilevel detection heads [9], and a modified YOLOv8 combines Bi-PAN-FPN with lightweight modules [10]. These methods show the value of shallow spatial information and multiscale interaction. However, the dense detectors built upon such representations generally still require post-processing.

DETR formulates object detection as a set prediction problem and directly outputs a fixed number of object predictions through a Transformer encoder–decoder architecture with bipartite matching [11]. Building upon DETR, Deformable DETR reduces computational cost and improves small-object representation through multiscale deformable attention [12]. DAB-DETR introduces dynamic anchor boxes to provide explicit spatial priors for object queries [13], while DINO further improves detection accuracy and training stability through denoising training and hybrid query selection [14]. Efficient DETR initializes object queries using dense encoder predictions [15]. RT-DETR further achieves real-time end-to-end detection by introducing an efficient hybrid encoder together with uncertainty-minimal query selection [16], and RT-DETRv2 further improves model flexibility and practical performance through optimized multiscale sampling and training strategies [17].

For UAV object detection, Drone-DETR incorporates shallow $P_{2}$ features and a multipath fusion module into the RT-DETR framework [18]. D^2^-DETR enhances feature representation in complex scenes through joint frequency-spatial modeling [19], while BAP-DETR improves multiscale representation using attention mechanisms and a dual-fusion encoder [20]. Existing studies have primarily focused on improving detection performance through backbone design, encoder optimization, or feature fusion. However, few methods jointly exploit class-agnostic target priors for multiscale feature recalibration, shallow-detail enhancement, and query initialization. In densely populated scenes containing numerous small objects, selecting a fixed number of queries solely according to encoder classification responses may still result in insufficient candidate coverage.

Based on these observations, we propose TQ-DETR, a target-guided dynamic query framework built upon RT-DETRv2-R18. The proposed framework is centered on three key components: target-oriented spatial priors, dynamic query initialization, and shallow boundary enhancement. Rather than optimizing a single detection scale, TQ-DETR aims to improve candidate allocation and multiscale feature representation in complex UAV scenarios while preserving the efficiency of end-to-end detection. The main contributions of this work are summarized as follows:

(1) We introduce a class-agnostic target-oriented objectness prior that provides spatial guidance before category prediction, enabling unified optimization of feature representation and query selection.
(2) We develop a DQ-style dynamic query allocation strategy that preserves high-confidence queries generated by the RT-DETRv2 encoder while supplementing them with dynamically selected objectness-guided queries, thereby improving candidate coverage and query initialization quality.
(3) We propose OG-MBF, a target-oriented $P_{2}$-to-$P_{3}$ multiscale boundary fusion module. Guided by the class-agnostic target prior, OG-MBF selectively filters shallow boundary details through local-context modeling and high-frequency edge enhancement before injecting them into $P_{3}$ via residual connections, thereby providing richer localization cues for small objects without increasing the number of Transformer feature levels.

## 2. Related Work

### 2.1. UAV Object Detection

UAV object detection is particularly challenging because aerial images usually contain small and densely distributed objects, substantial scale variation, and complex background interference. Existing studies have addressed these challenges mainly through region-oriented processing and enhanced multiscale representation. Region-oriented approaches reduce redundant computation by concentrating detection resources on potentially informative areas. Representative methods include ClusDet, which performs local detection on clustered object regions [2], ESOD, which combines target-oriented region search with feature-level slicing [3], and QueryDet, which employs sparse computation guided by low-resolution features [4]. Localization-oriented methods such as NWD further improve small-object detection by introducing more suitable bounding-box similarity measures [5]. Although these approaches improve computational allocation or localization for small targets, they generally rely on specialized region-processing or regression strategies.

Another major direction focuses on preserving fine-grained information during multiscale feature extraction. FPN and EFPN establish hierarchical feature interaction and high-resolution texture propagation [6,7], while UAV-oriented detectors such as TPH-YOLOv5, Drone-YOLO, and improved YOLOv8 variants enhance shallow features, introduce additional detection scales, or strengthen bidirectional feature aggregation [8,10]. Recent end-to-end UAV detectors have further incorporated shallow-feature fusion and specialized feature enhancement into Transformer-based architectures [18,20]. ESO-DETR further enhances small-object representation in UAV imagery through attention-based feature extraction and multiscale feature fusion within the RT-DETR framework [21]. These studies demonstrate the importance of high-resolution spatial information for detecting small aerial objects. However, shallow representations also contain abundant background textures and high-frequency interference. Therefore, effectively exploiting fine-grained information while suppressing irrelevant background responses remains an important issue in UAV object detection.

### 2.2. DETR-Based Object Detection

DETR reformulates object detection as a set prediction problem and simplifies the conventional detection pipeline through bipartite matching. Despite its elegant framework, DETR suffers from slow convergence and high computational cost when processing high-resolution feature maps [11]. Deformable DETR significantly reduces the computational burden by introducing multiscale deformable attention with a limited number of learnable sampling points [12]. DAB-DETR further incorporates dynamic anchor boxes into decoder queries, providing explicit spatial priors for object representation [13], while DINO improves both query quality and training stability through denoising training and hybrid query selection [14]. Efficient DETR demonstrates that high-quality query initialization can effectively reduce the iterative burden of the decoder by generating object queries from dense encoder predictions [15].

RT-DETR extends DETR to real-time object detection by introducing an efficient hybrid encoder together with uncertainty-minimal query selection [16]. RT-DETRv2 further improves multiscale sampling, data augmentation strategies, and deployment efficiency [17]. However, its query initialization mechanism still primarily relies on encoder classification responses. In UAV imagery, distant, heavily occluded, or low-contrast objects often produce relatively weak classification responses, making them less likely to be selected as initial decoder queries. This limitation suggests that category-independent foreground cues could provide complementary spatial evidence for query initialization, particularly for small objects with weak semantic responses.

### 2.3. Object-Oriented Feature Enhancement and Query Initialization

Feature representation and query initialization affect different stages of end-to-end detection but are closely related in UAV imagery. Multiscale and shallow-feature enhancement methods preserve spatial details that are easily degraded by repeated downsampling [6,7,18], whereas object-query optimization methods improve the quality of candidates entering the Transformer decoder [13,16]. These two directions are particularly important for small aerial objects, whose limited spatial extent can simultaneously weaken feature responses and reduce their probability of being selected as decoder candidates.

Nevertheless, the two problems are generally addressed independently. Directly incorporating shallow features can introduce substantial background textures together with useful boundary information, while query initialization based primarily on category confidence may overlook objects with weak semantic responses. A spatial prior indicating the likelihood of foreground objects could therefore serve as a bridge between these two stages: it can help distinguish target-related fine-grained information from background interference during feature enhancement and provide complementary localization evidence during query selection.

Motivated by these limitations, we investigate a unified target-oriented framework in which class-agnostic objectness cues provide complementary spatial guidance for both feature enhancement and query initialization. This design aims to preserve target-relevant fine-grained information while improving candidate coverage for objects with weak semantic responses.

## 3. Our Method

### 3.1. Overall Network Architecture

Building upon the RT-DETRv2-R18 detector, we propose TQ-DETR for small-object detection in UAV remote sensing imagery. Given an input image (I), the ResNet-18 backbone extracts four stages of feature maps, namely $P_{2}$, $P_{3}$, $P_{4}$, and $P_{5}$, with strides of 4, 8, 16, and 32 and channel dimensions of 64, 128, 256, and 512, respectively. Among them, $P_{2}$ preserves high-resolution spatial information, including object boundaries, corner structures, and local textures, whereas $P_{3}$–$P_{5}$ progressively encode richer semantic and contextual representations through successive downsampling. As illustrated in Figure 1, the proposed framework consists of six key components: target-oriented $P_{2}$-to-$P_{3}$ boundary fusion, TOB-based objectness modeling, multiscale feature recalibration, a hybrid encoder, dynamic query initialization, and a Transformer decoder.

TQ-DETR employs $P_{2}$ as a dedicated detail enhancement branch and injects shallow features into the $P_{3}$ stage. For an input resolution of 640 × 640 for example, $P_{2}$ contains 160 × 160 = 25,600 spatial locations, whereas the combined feature maps of $P_{3}$, $P_{4}$, and $P_{5}$ contain 80 × 80 + 40 × 40 + 20 × 20 = 8400 locations. Directly incorporating $P_{2}$ into the Transformer’s encoding memory would increase the sequence length to 34,000, approximately 4.05 times that of the original three-scale representation, resulting in substantially higher memory consumption and computational cost for multiscale attention. To avoid this issue, OG-MBF first performs channel projection and scale alignment on $P_{2}$, and then generates spatial gating weights by jointly exploiting semantic information from $P_{3}$ and class-agnostic target priors. The selected boundary features are subsequently fused into $P_{3}$ to produce the enhanced feature map $P_{3}^{*}$. The subsequent encoder and decoder operate on $F_{3}^{\prime }$, $F_{4}^{\prime }$, and $F_{5}^{\prime }$, thereby preserving the original three-scale encoding structure while incorporating fine-grained information from $P_{2}$.

The TOB operates in two sequential stages. First, its lightweight objectness predictor generates an initial objectness response from the original $P_{3}$ feature map. This response, together with the $P_{2}$ boundary representation and $P_{3}$ semantic features, is used to guide the gating process of OG-MBF, thereby determining where and to what extent shallow boundary information should be injected. Subsequently, the parameter-shared predictor generates a refined objectness probability map M from the enhanced feature map $P_{3}^{*}$. After scale alignment, M is employed to perform residual target-guided recalibration of $P_{3}^{*}$, $P_{4}$, and $P_{5}$. Following feature flattening and index alignment, the same objectness map also provides class-agnostic spatial candidates for dynamic query initialization. The two objectness predictions serve different purposes: the first guides boundary filtering before feature fusion, whereas the second supports feature recalibration and query generation after feature enhancement. Sharing parameters between the two prediction stages effectively limits the additional model complexity.

The recalibrated feature maps $F_{3}^{\prime }$, $F_{4}^{\prime }$, and $F_{5}^{\prime }$ are projected to a common feature dimension of 256 through 1 × 1 convolutions before entering the RT-DETRv2 hybrid encoder. The encoder performs intra-scale interaction on high-level representations and cross-scale aggregation through top-down FPN and bottom-up PAN pathways, producing three encoded feature memories, $E_{3}$, $E_{4}$, and $E_{5}$. This design retains the feature hierarchy and strides of the original encoder and allows the proposed modules to be integrated without changing the three-scale feature interface.

During query initialization, the encoder classification head predicts classification responses for every spatial location, while the objectness probability map provides complementary class-agnostic foreground responses aligned with the encoded features. The total number of decoder queries is fixed at 400. Among them, 300 queries are selected according to encoder classification scores, whereas the remaining queries are supplemented using high-ranking objectness responses. The two candidate sets are concatenated and deduplicated while preserving their original ranking order. If duplicate indices result in fewer than 400 valid candidates, the remaining query slots are filled using the next highest semantic responses to maintain a fixed query budget. Based on the final query indices, the corresponding query embeddings and initial reference boxes are extracted from the encoded feature memory E and candidate box set B before being fed into a three-layer Transformer decoder. Each decoder layer progressively refines object representations through query self-attention, multiscale deformable cross-attention, and feed-forward networks, while the classification and bounding-box regression heads generate the final detection results. During training, the model is optimized using bipartite matching and set prediction loss, whereas no non-maximum suppression (NMS) is required during inference.

### 3.2. Target-Oriented Branch (TOB) and Multi-Scale Feature Recalibration

Small objects in UAV imagery typically occupy only a limited number of pixels. After successive downsampling operations, their category-specific appearance, texture, and semantic information are progressively weakened, whereas relatively stable structural cues around object centers can still be preserved. Since the encoder classification responses are jointly influenced by object scale, class distribution, and background interference, they cannot always accurately reflect the likelihood of foreground object existence. To address this limitation, we introduce a TOB-based objectness modeling strategy that learns a spatial probability map representing foreground object likelihood independent of category semantics. This objectness map serves as a shared spatial prior for both multiscale feature enhancement and dynamic query initialization without performing category prediction.

TOB operates on the enhanced feature map $P_{3}^{*}$ generated by OG-MBF. Compared with deeper feature levels, $P_{3}$ provides a more favorable balance between spatial resolution and semantic representation, making it well suited for modeling small-object distributions. Specifically, its objectness predictor first employs a 13 × 13 depthwise convolution to aggregate local contextual information, followed by batch normalization, a ReLU activation, and a 1 × 1 convolution for channel aggregation. Let Z denote the objectness logits and M denote the corresponding objectness probability map. The logits generation and probability mapping are formulated as shown in Equations (1) and (2).

(1) $$Z={\mathrm{Conv}}_{1\times 1}(\mathrm{ReLU}(\mathrm{BN}({\mathrm{DWConv}}_{13\times 13}(P_{3}^{*}))))$$

(2) M=σ(Z)

where Z and M have dimensions of $B\;\times \;1\;\times \;H_{3}\;\times \;W_{3}$, and each element in M represents the probability that the corresponding spatial location belongs to a foreground object regardless of its category. The large-kernel depthwise convolution effectively enlarges the receptive field while introducing only limited computational overhead, enabling the network to capture local structural cues surrounding object centers. Channel aggregation further removes category-specific information, allowing objects from different categories to jointly contribute to learning a unified foreground representation. Consequently, M provides complementary spatial evidence for object localization, particularly in regions where classification responses are relatively weak.

During training, Gaussian soft labels are generated according to the centers of the ground-truth bounding boxes, and the responses of overlapping objects are merged using a maximum operation. Compared with conventional rectangular supervision, Gaussian center supervision places greater emphasis on stable object centers and is better aligned with the initialization requirements of decoder reference points. The objectness branch is optimized using a combination of soft focal binary cross-entropy loss and Dice loss. The former alleviates the severe imbalance between foreground and background samples, while the latter further enhances supervision on sparse foreground regions from the perspective of regional overlap.

After obtaining the objectness probability map, the learned spatial prior is propagated to $P_{3}^{*}$, $P_{4}$, and $P_{5}$ for multi-scale feature enhancement. Since different feature levels have different spatial resolutions, M is first resized by bilinear interpolation and then broadcast along the channel dimension. The residual feature recalibration process is formulated as shown in Equations (3) and (4).

(3) $$\alpha_{k}=\alpha_{max}\sigma (u_{k})$$

(4) $$F_{k}^{\prime }=F_{k}\;⊙\;[1+\alpha_{k}R_{k}(M)],\;k\;\in \;\{3,\;4,\;5\},\;\alpha_{max}=0.2$$

where $R_{k}(\cdot )$ denotes the scale-alignment and channel-broadcasting operation for the k-th feature level. $u_{k}$ is a learnable scale-specific control parameter, and $\alpha_{k}$ is the corresponding residual gain after sigmoid mapping. Following the moderate residual-scaling range from 0.1 to 0.3 reported by Szegedy et al. [22], $\alpha_{max}$ is set to 0.2 as a conservative upper bound. Since $R_{k}(M)$ takes values between 0 and 1, the multiplicative factor in Equation (4) remains below 1.2, preventing the objectness prior from excessively perturbing the original features. When $u_{k}$ is initialized to zero, $\sigma (u_{k})$ = 0.5 and the initial value of $\alpha_{k}$ is therefore 0.1. During training, $\alpha_{k}$ is independently optimized for each feature level within the prescribed range.

Residual recalibration preserves the original feature representations while selectively enhancing regions with high foreground probability through moderate residual amplification. As a result, background context and low-response regions remain available for subsequent feature encoding. The recalibrated feature maps retain the original channel dimensions and spatial resolutions and can therefore be directly fed into the RT-DETRv2 hybrid encoder without introducing additional architectural modifications. During training, auxiliary supervision enables the objectness branch to gradually learn stable foreground priors. During inference, the same objectness map simultaneously guides multiscale feature recalibration and provides spatial candidates for the dynamic query initialization strategy described in Section 3.3. Consequently, the proposed objectness branch establishes a unified information flow connecting feature enhancement with query initialization.

Figure 2 illustrates the overall workflow of TOB and multiscale feature recalibration. As shown, TOB predicts a single-channel objectness probability map from the enhanced $P_{3}^{*}$ feature map. The resulting objectness map is subsequently resized to different feature scales through bilinear interpolation, broadcast across feature channels, and incorporated into $P_{3}^{*}$, $P_{4}$, and $P_{5}$ via constrained residual recalibration. This mechanism selectively enhances potential foreground regions while suppressing excessive amplification of background responses, thereby providing more discriminative multiscale feature representations for the subsequent hybrid encoder.

### 3.3. Objectness-Guided Dynamic Query Initialization

RT-DETRv2 initializes decoder queries by selecting the Top-*K* spatial locations according to the maximum classification responses predicted by the encoder. Although this strategy effectively preserves high-confidence candidates, it tends to favor large objects, salient background regions, or a few dominant categories in densely populated UAV scenes. Consequently, many small objects with relatively weak classification responses may fail to enter the decoding stage. To alleviate this limitation, the proposed objectness probability map provides complementary foreground localization cues that are independent of semantic classification. By jointly exploiting semantic confidence and objectness responses, we design an objectness-guided dynamic query initialization strategy that allocates the query budget to both semantic candidates and objectness-guided candidates within a fixed number of decoder queries.

Assume that the encoded feature memories from $F_{3}^{\prime }$, $F_{4}^{\prime }$, and $F_{5}^{\prime }$ are flattened and concatenated into a unified feature sequence containing N spatial locations. The encoder classification head predicts classification logits for each location, and the semantic score vector s is obtained by selecting the maximum classification response at every position. Meanwhile, the objectness probability map M is resized to the corresponding feature resolutions, flattened following the same ordering, and concatenated into an objectness score vector m. Consequently, each element in s and m corresponds to the same spatial position within the encoded feature sequence.

The semantic score reflects the confidence of category discrimination, whereas the objectness score estimates the probability that a location belongs to a foreground object regardless of category. Since these two scores represent different objectives, directly combining them through weighted summation is highly sensitive to score distributions and weighting coefficients. Moreover, locations associated with weak semantic responses may still be suppressed during global ranking. Instead, the proposed method explicitly allocates separate query budgets to semantic candidates and objectness-guided candidates. Semantic ranking preserves locations with reliable category predictions, while objectness ranking supplements previously unselected locations exhibiting strong foreground responses, thereby providing additional opportunities for potential small objects to participate in subsequent decoding.

Let B denote the set of inactive candidate boxes, and let $N_{b}$, $N_{d}$, and $N_{q}$ denote the numbers of semantic candidates, objectness-guided candidates, and total decoder queries, respectively. The dynamic query initialization process consists of candidate selection, index deduplication and budget completion, and query-feature extraction, as formulated in Equations (5)–(7).

(5) $$J_{b}=\mathrm{TopK}(s,\;N_{b}),\;J_{d}=\mathrm{TopK}(m,\;N_{d})$$

(6) $$J_{q}={\mathrm{Fill}}_{N_{q}}(\mathrm{Dedup}(J_{b}\;∥\;J_{d});\;s)$$

(7) $$Q_{0}=\mathrm{Gather}(E,\;J_{q}),\;R_{0}=\mathrm{Gather}(B,\;J_{q})$$

where ∥ denotes ordered concatenation. Dedup(·) removes repeated indices while retaining their first occurrence, and ${\mathrm{Fill}}_{N_{q}}$(·) completes the candidate set to $N_{q}$ using the highest-scoring unused indices from s. Because $J_{b}$ precedes $J_{d}$, the procedure preserves semantic candidates when the two Top-K sets overlap and appends only nonduplicate objectness-guided candidates. We set $N_{b}$ = 300, $N_{d}$ = 100, and $N_{q}$ = 400. The overlap between the two sets determines the number of retained objectness-guided candidates, while the decoder always receives 400 queries.

Using the final indices $J_{q}$, Gather extracts the query embeddings $Q_{0}$ and initial reference boxes $R_{0}$ from the encoded feature memory E and candidate box set B, respectively. OG-MBF uses the $P_{2}$ feature only for shallow boundary enhancement; neither the encoder memory nor the decoder query set includes $P_{2}$. The decoder therefore retains the original three-scale encoded representation for cross-attention. We detach $Q_{0}$ and $R_{0}$ before decoding so that gradients from the discrete selection process do not propagate into the encoder, while the auxiliary detection branches continue to supervise the encoder classification and regression heads.

The proposed strategy modifies the composition of the decoder queries while maintaining a fixed query budget, leaving the decoder architecture and inference procedure unchanged. By explicitly allocating query resources to objectness-guided candidates, the method reduces its dependence on classification confidence alone and improves candidate coverage for densely distributed small objects.

Figure 3 illustrates the objectness-guided dynamic query initialization process. The method first retains the 300 highest-ranked semantic candidates from RT-DETRv2 and then adds up to 100 previously unselected objectness-guided candidates through deduplication and budget completion. The resulting 400-query set allows small objects with weak semantic responses but distinct foreground structure to enter the decoder.

### 3.4. Target-Oriented $P_{2}$-to-$P_{3}$ Multiscale Boundary Fusion

Repeated downsampling inevitably weakens the boundary and localization cues of small objects, whereas the shallow $P_{2}$ feature map preserves abundant high-resolution information, including object boundaries, corner structures, and fine textures. However, $P_{2}$ also contains considerable high-frequency background noise, such as road markings, building contours, and shadow boundaries. Directly adding or concatenating $P_{2}$ with $P_{3}$ inevitably introduces substantial background interference into the semantic features. Alternatively, treating $P_{2}$ as an additional Transformer feature level would considerably increase the computational burden of the encoder. To address these issues, we propose OG-MBF, a target-oriented $P_{2}$-to-$P_{3}$ multiscale boundary fusion module that extracts, filters, and injects shallow boundary information entirely within the convolutional domain. As illustrated in Figure 4, OG-MBF consists of three sequential stages: $P_{2}$ boundary representation learning, target-guided spatial gating, and residual boundary injection into $P_{3}$.

First, a 1 × 1 convolution projects the original 64-channel $P_{2}$ feature map into a 128-dimensional hidden feature space. The boundary extraction branch consists of three parallel pathways, namely a local feature branch, a contextual feature branch, and an edge enhancement branch. The local branch employs a 3 × 3 depthwise convolution followed by a 1 × 1 pointwise convolution to capture local texture information. The contextual branch adopts a 3 × 3 depthwise convolution with a dilation factor of 2 to enlarge the receptive field and capture broader contextual information. The edge branch extracts high-frequency residual information by subtracting the output of 3 × 3 average pooling from the original feature map and subsequently predicts a single-channel edge attention map. The projection, high-frequency residual extraction, edge-aware fusion, and scale alignment are formulated as shown in Equations (8)–(10).

(8) $$X=\varphi_{p}\left(P_{2}\right),\;D=X-{\mathrm{AvgPool}}_{3\times 3}\left(X\right)\;$$

(9) $$B=[\varphi_{loc}(X)+{\varphi_{ctx}}^{d}(X)]\;⊙\;[1+\sigma (\varphi_{e}(D))]$$

(10) $$B_{3}=\varphi_{down}(B)$$

where φ_p denotes the channel projection of P_2, D represents the high-frequency residual, φ_loc and φ_ctx^d correspond to the local and contextual transformation functions, respectively, and φ_e denotes the edge attention predictor. The local branch captures fine-grained texture and boundary cues, whereas the contextual branch employs dilated depthwise convolution to incorporate a broader receptive field and provide complementary contextual information. Their representations are first fused through element-wise addition and subsequently modulated by the edge attention map using a residual multiplication strategy. In this manner, non-edge regions preserve their original responses, while spatial locations containing prominent high-frequency structures receive additional enhancement. Finally, the downsampling operator φ_down, consisting of a 3×3 convolution with stride 2 followed by batch normalization and ReLU activation, aligns the boundary representation with the spatial resolution of P_3, yielding the boundary feature B_3.

Although B_3 preserves fine-grained boundary information from the shallow feature P_2, edge information alone may be insufficient to distinguish true object boundaries from complex background textures, such as road markings, building contours, and shadow boundaries. Therefore, OG-MBF further incorporates semantic information and class-agnostic objectness guidance to perform target-aware boundary selection. Specifically, the shared TOB predictor first generates an initial objectness map M^(0) from the original feature map P_3, providing a coarse spatial prior that indicates the likelihood of foreground objects before boundary fusion. Meanwhile, P_3 is projected through a 1×1 convolution to obtain the semantic representation S_3. The semantic feature S_3, the aligned boundary representation B_3, and the initial objectness prior M^(0) are then concatenated along the channel dimension and fed into a lightweight gating network to generate a single-channel spatial gate G. Guided jointly by semantic context and class-agnostic foreground cues, G selectively retains target-relevant boundary responses while suppressing high-frequency structures arising from irrelevant background regions. The filtered boundary representation is subsequently refined by a 3×3 convolution and injected into P_3 through a learnable residual connection, producing the enhanced feature map P_3^∗, as formulated in Equations (11)–(13).

(11) $$M^{\left(0\right)}=\sigma (\mathrm{TOB}(P_{3}))$$

(12) $$G=\sigma (\varphi_{g}([\varphi_{3}(P_{3}),\;B_{3},\;M^{\left(0\right)}]))\;$$

(13) $${P_{3}}^{*}=P_{3}+\gamma \varphi_{r}(G\;⊙\;B_{3})$$

Here, $\varphi_{3}$ denotes the semantic projection of $P_{3}$, $\varphi_{g}$ represents the gating network consisting of a 1×1 convolution, batch normalization, ReLU activation, another 1×1 convolution, and a sigmoid activation, whereas $\varphi_{r}$ denotes the refinement convolution. The learnable residual coefficient γ is initialized to 0.1. This setting follows the small-value initialization principle used for learnable residual scaling in LayerScale [23], which limits the contribution of the boundary branch at the beginning of training and allows the model to incorporate the additional boundary information progressively. Since the gating network simultaneously exploits high-level semantic representations, shallow boundary information, and class-agnostic objectness priors, the resulting spatial gate performs target-aware boundary selection rather than conventional edge enhancement. The residual formulation further ensures that the original semantic representation dominates the early stage of training while allowing the network to learn an appropriate amount of boundary information to inject.

It is worth emphasizing that the objectness prior $M^{\left(0\right)}$ is exclusively used to guide boundary filtering before feature fusion, whereas the refined objectness map described in Section 3.2 is predicted again from the enhanced feature map $P_{3}^{*}$ and subsequently employed for feature recalibration and dynamic query initialization. Although both prediction stages share the same TOB parameters, they perform different functions within a sequential processing pipeline. Specifically, the original $P_{3}$ first provides coarse objectness guidance for boundary filtering, OG-MBF subsequently enhances shallow boundary representations, and the refined $P_{3}^{*}$ then generates a more accurate objectness map for subsequent feature enhancement and query initialization. This design establishes a clear information dependency between boundary enhancement and dynamic query generation without introducing recursive feature refinement.

Figure 4 illustrates the complete information flow within OG-MBF. The shallow $P_{2}$ feature map first undergoes channel projection, local texture extraction, contextual aggregation, and edge enhancement to generate a discriminative boundary representation. After spatial alignment, the boundary representation is jointly processed with the semantic projection of $P_{3}$ and the initial objectness prior through the target-guided gating module. The filtered boundary information is finally injected into $P_{3}$ via a residual connection, thereby providing richer localization cues while effectively suppressing irrelevant background texture.

The output of OG-MBF has the same spatial resolution and channel dimension as $P_{3}$. $P_{2}$ is processed only by convolutional operations and one downsampling stage; it is not added as a high-resolution Transformer feature level. OG-MBF therefore preserves the original three-scale encoder interface. Its computational cost, together with the cost of the other proposed components, is reported in the complexity analysis.

## 4. Experiments and Result Analysis

### 4.1. Dataset and Evaluation Metrics

Experiments were conducted on the VisDrone2019-DET benchmark dataset. The dataset was collected using UAV platforms from diverse viewpoints and under varying environmental conditions and is widely used for evaluating object detection algorithms in aerial imagery. It contains 8629 images, including 6471 training images, 548 validation images, and 1610 testing images, accounting for approximately 75.0%, 6.4%, and 18.7% of the dataset, respectively. The images cover a variety of real-world scenarios, including urban roads, residential areas, public squares, and parking lots. The dataset contains multiple object categories, such as pedestrians, people, cars, vans, trucks, buses, motor, and bicycles, and is characterized by small object sizes, severe class imbalance, dense object distributions, and highly uneven spatial layouts.

Figure 5 summarizes the statistical characteristics of the training annotations. Figure 5a reveals a pronounced class imbalance, with cars and pedestrians accounting for a large proportion of the annotated instances, whereas tricycles, awning-tricycles, and buses are relatively underrepresented. According to the COCO scale criteria, small objects account for 61.5% of all annotated instances (Figure 5b). As shown in Figure 5c, each training image contains an average of 48.42 objects, with a maximum of 922 instances. The center-density map in Figure 5d further reveals a highly nonuniform spatial distribution of objects, with annotations concentrated in specific regions of the images.

These statistics confirm that VisDrone2019 combines a high proportion of small objects(defined as objects with a bounding-box area smaller than 32 × 32 pixels) with class imbalance, dense scenes, and uneven spatial layouts. It is therefore suitable for evaluating small-object detection in UAV imagery.

Following the COCO evaluation protocol, we report AP, AP_50_, AP_75_, AP_s_, AP_m_, AP_l_ and recall under different maximum detection counts. AP is averaged over intersection-over-union thresholds from 0.50 to 0.95; AP_50_ and AP_75_ use thresholds of 0.50 and 0.75. AP_s_, AP_m_ and AP_l_ measure performance on small, medium, and large objects, respectively. Because this study focuses on small objects, AP_s_ and small-object recall receive particular attention.

### 4.2. Experimental Setup

Unless otherwise specified, all experiments used an input resolution of 640 × 640 and a ResNet-18 backbone, and the models were trained for 160 epochs. TQ-DETR includes TOB, objectness-guided dynamic query initialization, and OG-MBF. It uses 400 decoder queries: 300 semantic queries selected from encoder classification responses and up to 100 objectness-guided queries derived from the objectness heatmap.

Only minimal cleaning was applied to anomalous annotations in the training set; the original validation and test sets were not modified. All ablation variants used the same dataset split, input resolution, training schedule, and evaluation protocol. Table 1 summarizes the hardware and software environment.

To assess the sensitivity of the main results to training randomness, RT-DETRv2-R18 and TQ-DETR were independently trained and evaluated using three random seeds (0, 1, and 2). For each run, the model was initialized from the same COCO-pretrained checkpoint, while the dataset split, annotations, input resolution, batch size, training schedule, optimizer, and evaluation protocol were kept unchanged. The results are reported as the mean ± sample standard deviation over the three runs. Unless otherwise specified, the ablation and sensitivity experiments were conducted using seed 0.

The core training settings are listed in Table 2; parameters not listed in the table retained their default values.

### 4.3. Comparison with Baseline Models and Existing Methods

We evaluate TQ-DETR from two perspectives. First, Table 3 compares it with the RT-DETRv2-R18 baseline using the same dataset split, input resolution, training schedule, and evaluation protocol. Second, Table 4 summarizes results reported for representative detectors on VisDrone2019-Val. Because those methods differ in architecture, training strategy, implementation, and measurement protocol, Table 4 provides context rather than a strictly controlled ranking. The main performance claims in this study are based on the controlled comparison in Table 3 and the ablations in Table 5.

As shown in Table 3, TQ-DETR achieved an AP of 30.21 ± 0.51%, compared with 28.42 ± 0.51% for RT-DETRv2-R18, corresponding to an average improvement of 1.79%. The mean AP50, AP75, and APs increased by 2.75%, 2.14%, and 1.81%, respectively. TQ-DETR outperformed the baseline under each of the three random seeds for all six reported metrics. The standard deviations did not exceed 0.74% for either model, indicating limited sensitivity to the evaluated random seeds under the current training protocol.

Table 4 provides a contextual comparison between TQ-DETR and representative CNN- and Transformer-based detectors reported on the VisDrone2019-DET validation set. TQ-DETR achieves an average AP of 30.21% and an average AP50 of 50.28%, with 20.51 M parameters and 69.38 G FLOPs. Compared with YOLOv12-M, which has a similar parameter count of 20.20 M, TQ-DETR improves AP and AP_50_ by 3.91% and 7.18%, respectively. It also achieves higher reported detection accuracy than conventional two-stage detectors and early DETR-based methods, including Faster R-CNN, DETR, and Deformable DETR, while requiring substantially fewer FLOPs than the latter two. Compared with RT-DETR-R50, TQ-DETR uses approximately 50.9% fewer parameters and 47.9% fewer FLOPs, although RT-DETR-R50 reports a higher AP. These results place TQ-DETR within a moderate model-complexity range while maintaining competitive reported detection performance.

Nevertheless, several recent UAV-oriented detectors report stronger accuracy–complexity trade-offs. In particular, VRF-DETR reports 31.80% AP and 51.40% AP_50_ with only 13.50 M parameters and 44.30 G FLOPs, showing better reported accuracy and lower computational complexity than TQ-DETR. More importantly, the methods summarized in Table 4 employ different architectures, training protocols, input configurations, and implementation settings; consequently, their reported results are provided as contextual cross-paper references rather than as a strictly controlled ranking.

Accordingly, the effectiveness of TQ-DETR is primarily evaluated through the controlled comparison with its direct baseline, RT-DETRv2-R18, in Table 3, where the dataset split, input resolution, training schedule, and evaluation protocol are kept identical. Under these conditions, TQ-DETR improves AP, AP_50_, AP_75_, and AP_s_ by 1.79%, 2.75%, 2.14%, and 1.81%, respectively. The improvement in AP_s_ indicates a measurable benefit for small-object detection, while the simultaneous gains in AP_50_ and AP_75_ suggest improved detection and localization under both relaxed and stricter IoU criteria. These controlled results support the effectiveness of the proposed target-oriented objectness modeling, dynamic query allocation, and boundary-aware feature fusion within the RT-DETRv2 framework.

### 4.4. Ablation Experiments

Table 5 reports ablations conducted under the same training settings. The baseline is RT-DETRv2-R18. TOB isolates the TOB; TOB + DQ adds objectness-guided query allocation; TOB + OG-MBF evaluates shallow detail fusion without DQ; and the full TQ-DETR model combines all three components.

Table 5 shows that TOB alone leaves AP essentially unchanged (27.84% to 27.80%), although AP_50_, AP_75_, and AP_s_ increase slightly. Adding DQ raises AP to 28.37% and AP_s_ to 19.45%, which indicates that the objectness prior is more useful when it participates in candidate allocation. The TOB + OG-MBF variant reaches 29.29% AP and 20.34% AP_s_, while the complete model further improves the overall AP to 29.53%. Notably, AP_l_ shows different trends across module combinations: TOB alone and TOB + OG-MBF achieve 57.99% and 57.66% AP_l_, respectively, whereas the complete model reaches 62.93%. This behavior reflects the complementary interaction between feature enhancement and query allocation. TOB and OG-MBF mainly strengthen foreground responses and boundary representations, whereas DQ determines how these enhanced target cues are converted into decoder candidates. By preserving high-confidence semantic queries while supplementing objectness-guided candidates, DQ enables the enhanced target regions produced by TOB and OG-MBF to participate more effectively in decoding. The query-allocation sensitivity results in Table 6 further support this interpretation, as the 300 + 100 configuration achieves the highest AP among the tested query ratios.

### 4.5. Parameter Sensitivity Analysis

To investigate the sensitivity of TQ-DETR to key hyperparameters and justify the default configuration used in the main experiments, we evaluate two parameters that directly affect the target-oriented query generation process. The first is the allocation ratio between semantic queries and objectness-guided queries under the fixed budget of 400 decoder queries. Semantic queries preserve candidates with reliable category responses, whereas objectness-guided queries provide complementary foreground candidates for objects with relatively weak classification confidence. Therefore, an appropriate balance between the two types of queries is required. The second parameter is the Gaussian standard deviation σ used to generate the soft objectness supervision in TOB. This parameter controls the spatial spread of the target-center response and consequently affects the learning of class-agnostic foreground priors.

As shown in Table 6, the 300 + 100 configuration achieves the best overall performance, reaching 29.53% AP, 49.49% AP_50_, 29.46% AP_75_, and 20.30% AP_s_. Compared with the 250 + 150 setting, AP and AP_s_ increase by 0.75% and 0.60%, respectively. Increasing the number of semantic queries to 350 while reducing objectness-guided queries to 50 also results in a lower AP of 28.88%. These results indicate that allocating too many objectness-guided queries may reduce the proportion of candidates with reliable semantic evidence, whereas an insufficient number of objectness-guided queries limits their ability to supplement small objects with weak classification responses. The 300 + 100 setting therefore provides a better balance between semantic reliability and foreground candidate coverage and is adopted in the remaining experiments.

Table 7 reports the sensitivity of the model to the Gaussian standard deviation used for objectness supervision. Among the tested values, σ = 2.0 produces the best results across all reported metrics, achieving 29.53% AP and 20.30% AP_s_. When σ is reduced to 1.0, AP decreases to 28.82% and AP_s_ to 19.55%, suggesting that an excessively concentrated Gaussian response provides limited spatial tolerance around target centers. In contrast, increasing σ to 3.0 broadens the supervision region and results in 28.97% AP and 19.72% AP_s_, indicating that overly diffuse supervision may weaken the spatial specificity of the objectness prior. Therefore, σ = 2.0 provides an appropriate balance between target-center concentration and spatial coverage and is used as the default setting.

### 4.6. Model Complexity and Latency Analysis

Table 8 compares the model complexity, inference speed, and detection performance of different configurations at an input resolution of 640 × 640. The reported AP, AP50, and APs values are the mean results over three independent runs, consistent with the evaluation protocol in Table 3. All inference-speed measurements were conducted under the same hardware, software, and inference settings to ensure a controlled comparison. Compared with RT-DETRv2-R18, the complete TQ-DETR increases the parameter count from 19.88 M to 20.51 M and the FLOPs from 61.19 G to 69.38 G, corresponding to increases of 3.2% and 13.4%, respectively. Meanwhile, AP, AP_50_, and AP_s_ increase by 1.79%, 2.75%, and 1.81%, respectively.

To further analyze the runtime contribution of the proposed components, we progressively introduced TOB, DQ, and OG-MBF under the same inference protocol. RT-DETRv2-R18, TOB, TOB + DQ, and the complete TQ-DETR achieve 81.39, 78.05, 59.84, and 52.88 FPS, corresponding to latencies of 12.29, 12.81, 16.71, and 18.91 ms per image, respectively. TOB introduces only a small latency increase, whereas the major runtime overhead occurs after introducing DQ, followed by OG-MBF.

The larger change in wall-clock speed relative to the 13.4% increase in FLOPs is mainly due to operations that are not fully reflected by conventional FLOPs. DQ increases the decoder query number from 300 to 400 and introduces Top-K ranking, candidate deduplication, feature gathering, and additional memory access, while OG-MBF adds shallow boundary extraction and feature fusion. Overall, TQ-DETR reduces the inference speed from 81.39 FPS to 52.88 FPS, corresponding to an approximately 35.0% decrease, while maintaining real-time inference capability on the evaluated GPU platform. Future work will focus on more efficient query processing, lightweight feature fusion, pruning, quantization, and other deployment-oriented optimizations.

### 4.7. Class-Level Performance and Small-Object Detection Analysis

Figure 6 compares the normalized confusion matrices of RT-DETRv2-R18 and TQ-DETR on the VisDrone2019 validation set, providing a class-level view of recognition and confusion patterns.

Table 9 compares the class-wise AP50 of TQ-DETR and RT-DETRv2-R18 for the seed-0 experiment. TQ-DETR improves the AP50 of all 10 categories, while the corresponding overall AP50 increases from 46.68% to 49.49%. Truck and pedestrian show gains of 4.2% and 4.0%, respectively, while bicycle and motor improve by 3.4% and 3.3%. These class-wise improvements are generally consistent with the confusion-matrix results in Figure 6. In particular, the diagonal responses of bicycle, motor, and tricycle increase from 0.32, 0.69, and 0.42 to 0.34, 0.70, and 0.47, respectively, while bicycle-to-motor confusion slightly decreases from 0.24 to 0.23. Nevertheless, bicycle and awning-tricycle remain among the most challenging categories, indicating that visual similarity among small objects is still an important source of classification error.

### 4.8. Visual Analysis

Figure 7 presents one representative dense UAV scene, together with the original image, confidence heatmaps, and detection overlays for RT-DETRv2-R18 and TQ-DETR. The example is intended to illustrate differences in missed detections, response concentration, and localization, rather than to serve as a comprehensive qualitative evaluation.

In the representative dense UAV scene shown in Figure 7, RT-DETRv2-R18 suffers from several missed detections and inaccurate localization in the crowded roadway region, especially for small objects with limited visual information. In contrast, TQ-DETR achieves more complete object coverage and generates more precise bounding boxes, demonstrating stronger detection capability in complex and densely distributed scenarios. This improvement benefits from the proposed target-guided query allocation strategy, which provides additional candidate opportunities for objects with weak semantic responses, and the boundary-aware feature fusion module, which enhances fine-grained spatial details for accurate localization. The qualitative results are consistent with the improvements in AP, AP_50_, and AP_s_ reported in Table 3, as well as the effectiveness of each proposed component verified in Table 5, further validating the capability of TQ-DETR for UAV small-object detection.

## 5. Discussion

Compared with the RT-DETRv2-R18 baseline, TQ-DETR consistently improves the reported detection metrics. AP_50_ and AP_75_ increase by 2.75% and 2.14%, respectively, indicating improved detection performance under both moderate and stringent IoU thresholds. AP_s_ increases from 19.25% to 21.06%, while AP_l_ increases from 58.81% to 62.79%. These results indicate that the proposed method improves detection across multiple object scales while maintaining its effectiveness for small-object detection. The three-seed experiments provide additional evidence for the reliability of the controlled comparison. Both models showed relatively small variation across seeds 0, 1, and 2, indicating limited sensitivity to random initialization under the evaluated training protocol. TQ-DETR also maintained a higher mean performance than RT-DETRv2-R18, suggesting that the observed improvement was not specific to a single training run.

The ablation results further demonstrate the complementary roles of the proposed components. Among the individual module variants, OG-MBF provides the largest AP improvement, highlighting the effectiveness of preserving shallow boundary and texture information for UAV small-object detection. The standalone TOB produces only a limited change in overall AP, suggesting that the objectness prior mainly serves as auxiliary target-aware guidance rather than an independent detection enhancement. Its effectiveness becomes more evident when the objectness prior is integrated into query allocation and feature fusion processes. Although the AP_s_ of the complete model (20.30%) is marginally lower than that of TOB + OG-MBF (20.34%), the difference is only 0.04%. Meanwhile, the complete model achieves higher overall AP, AP_m_, and AP_l_, and the query-allocation sensitivity analysis confirms that the 300 + 100 configuration provides the best performance among the tested ratios.

Several limitations remain. First, the current evaluation is conducted only on VisDrone2019, which limits the assessment of cross-dataset generalization. In addition, dedicated robustness evaluations under specific challenging UAV conditions, such as heavy occlusion, nighttime or low-illumination scenes, and substantial viewpoint, scale, or flight-altitude variations, have not yet been conducted. Second, although three independent runs indicate limited sensitivity to the selected random seeds, experiments with more seeds and additional datasets would provide a more comprehensive assessment of variability. Third, practical deployment on resource-constrained onboard UAV hardware has not yet been evaluated. Future work will therefore investigate broader UAV benchmarks, more challenging operating conditions, experiments with a larger number of random seeds, and deployment-oriented optimization, including model compression, pruning, and quantization.

## 6. Conclusions

This study proposed TQ-DETR, a target-guided end-to-end detector based on RT-DETRv2 for dense small-object detection in UAV remote sensing imagery. The proposed framework introduces a class-agnostic objectness modeling branch, an objectness-guided dynamic query allocation strategy, and an Objectness-Guided Multiscale Boundary Fusion (OG-MBF) module. These components enhance spatial prior modeling, query candidate coverage, and fine-grained feature representation within the DETR framework.

Experiments on the VisDrone2019-Val benchmark demonstrate that TQ-DETR consistently improves the detection performance of RT-DETRv2-R18, particularly for small-object detection. Ablation experiments further verify the complementary contributions of the proposed components, while the overall performance improvement is achieved with a moderate computational cost.

Overall, TQ-DETR provides an effective approach for improving RT-DETRv2-based detection on the evaluated VisDrone2019 benchmark. Future work will focus on improving inference efficiency and evaluating the generalization capability of the proposed framework across diverse UAV datasets and real-world deployment scenarios.

## Figures and Tables

**Figure 1:**
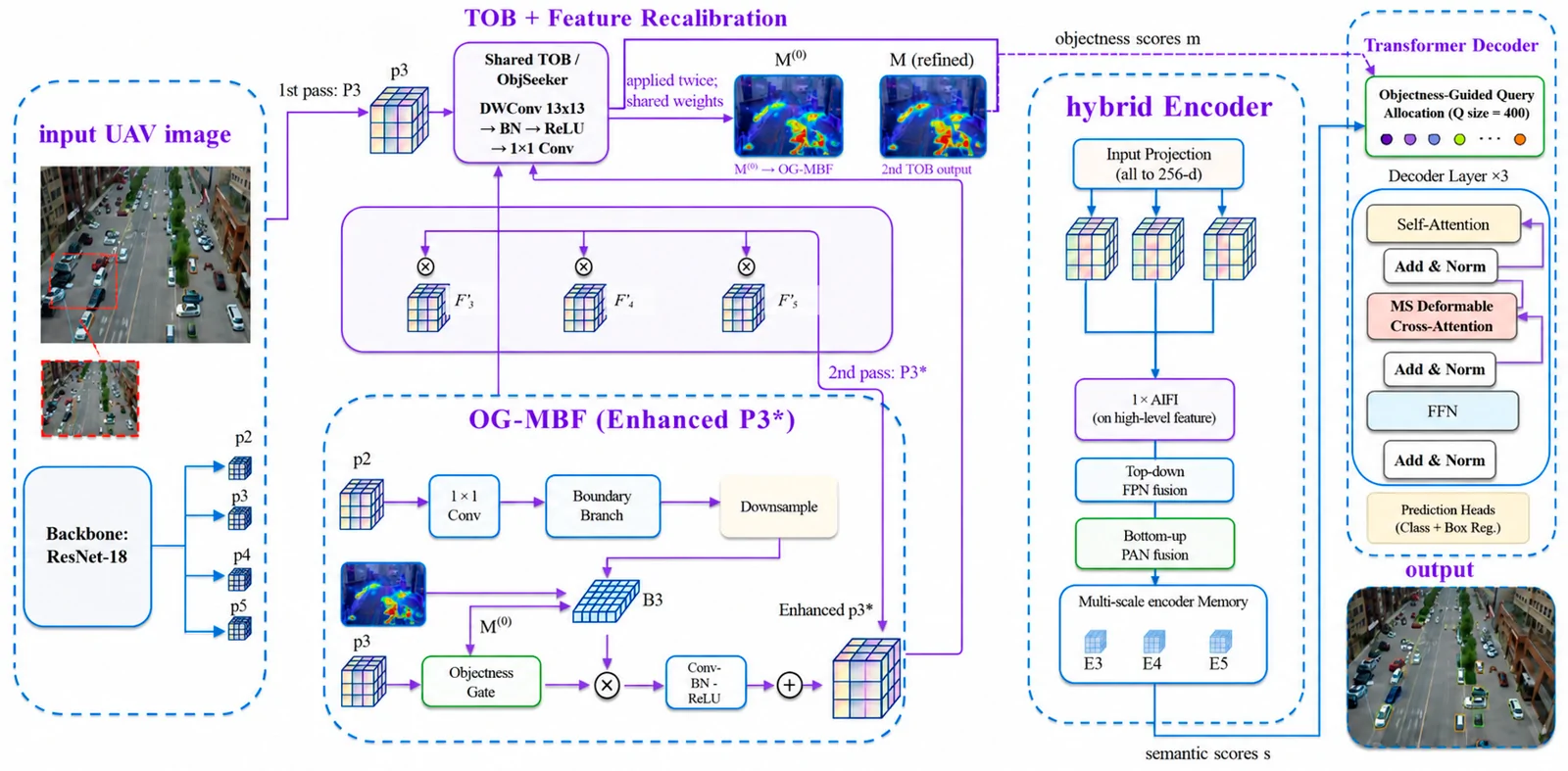
Overall architecture of TQ-DETR.

**Figure 2:**
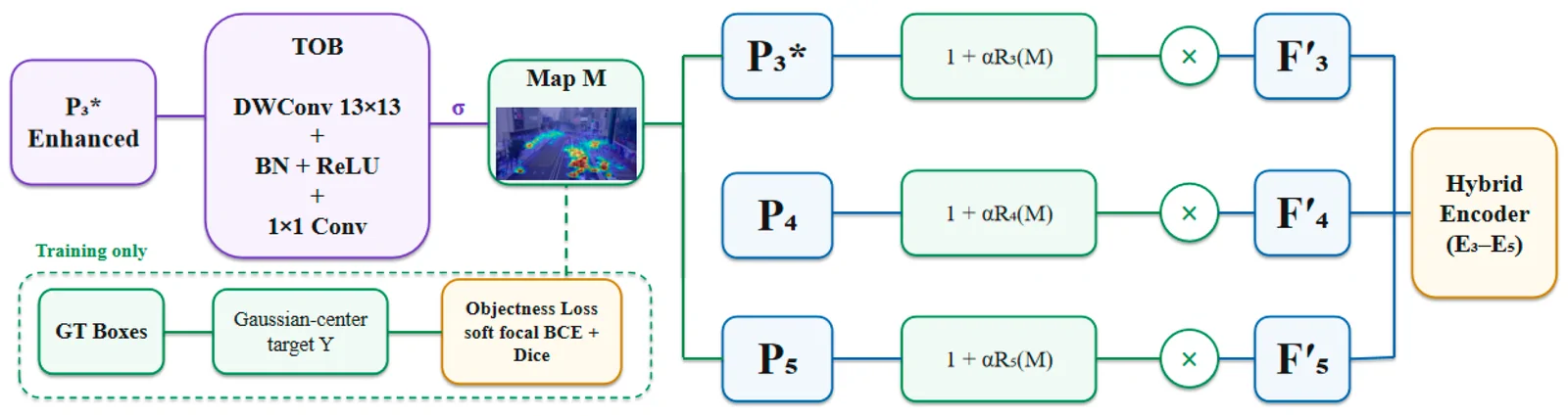
TOB and multiscale residual recalibration.

**Figure 3:**
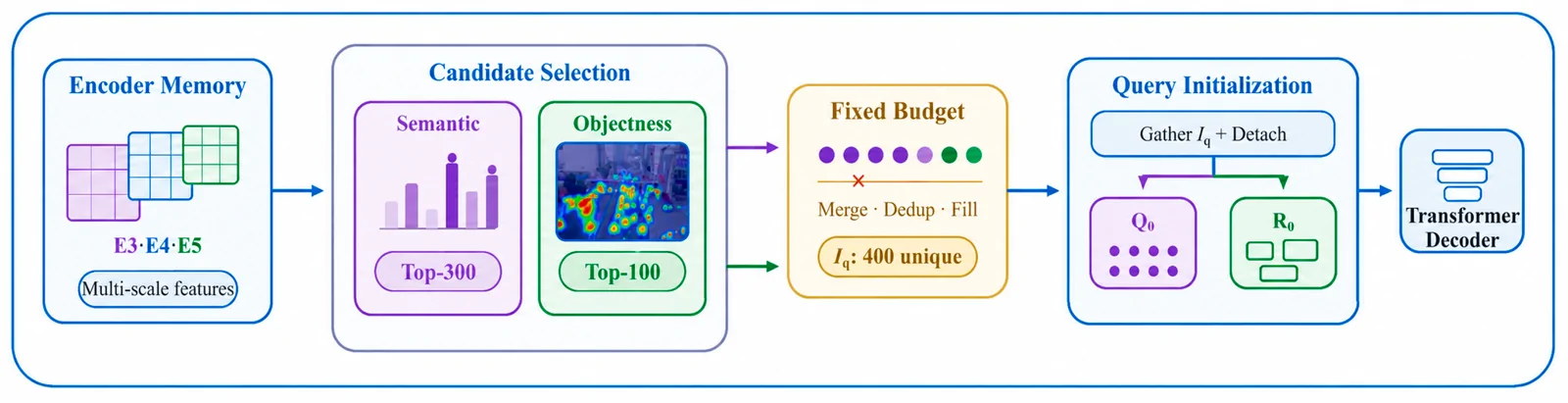
Dynamic query construction.

**Figure 4:**
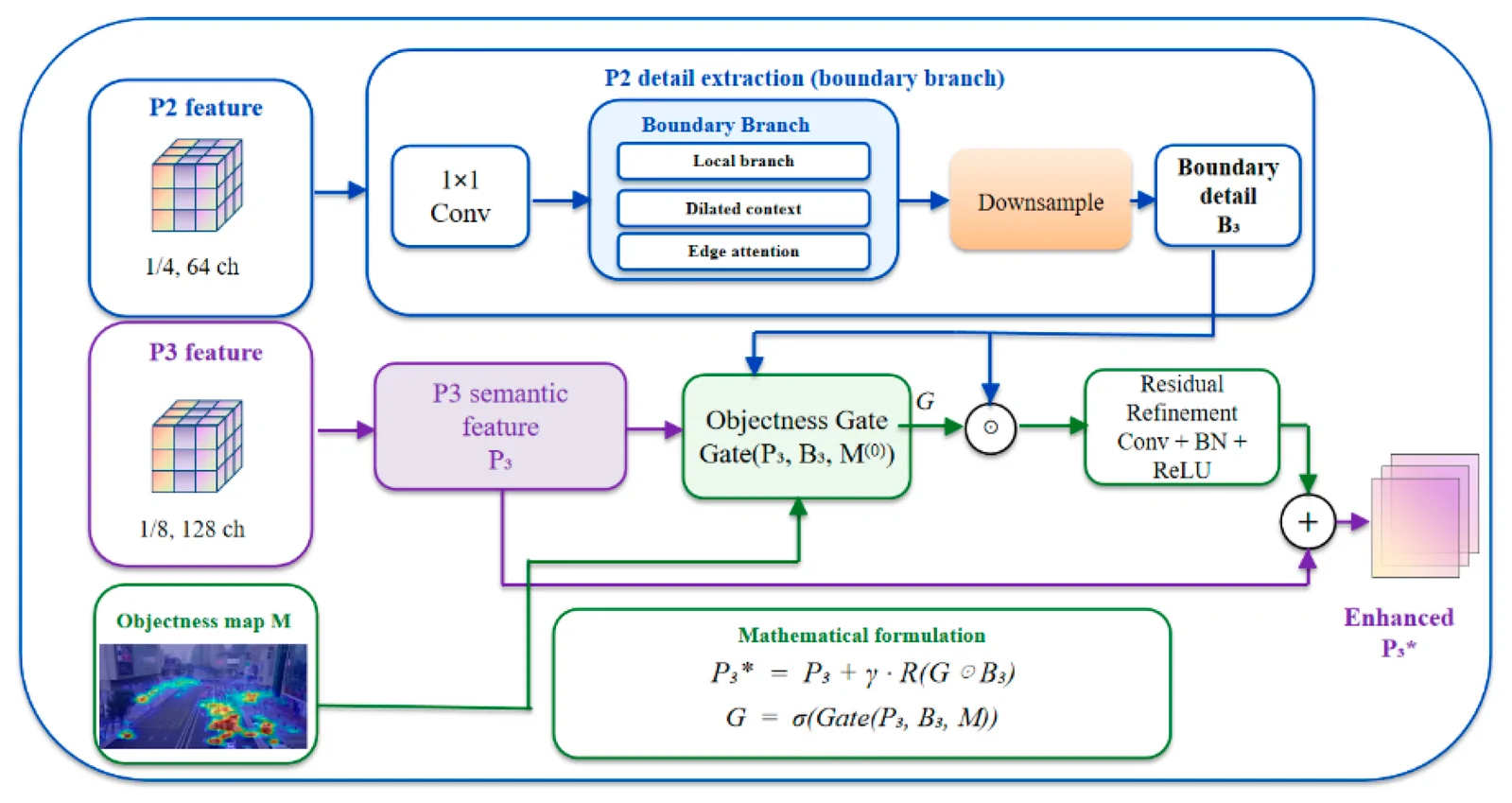
Structure of the OG-MBF module.

**Figure 5:**
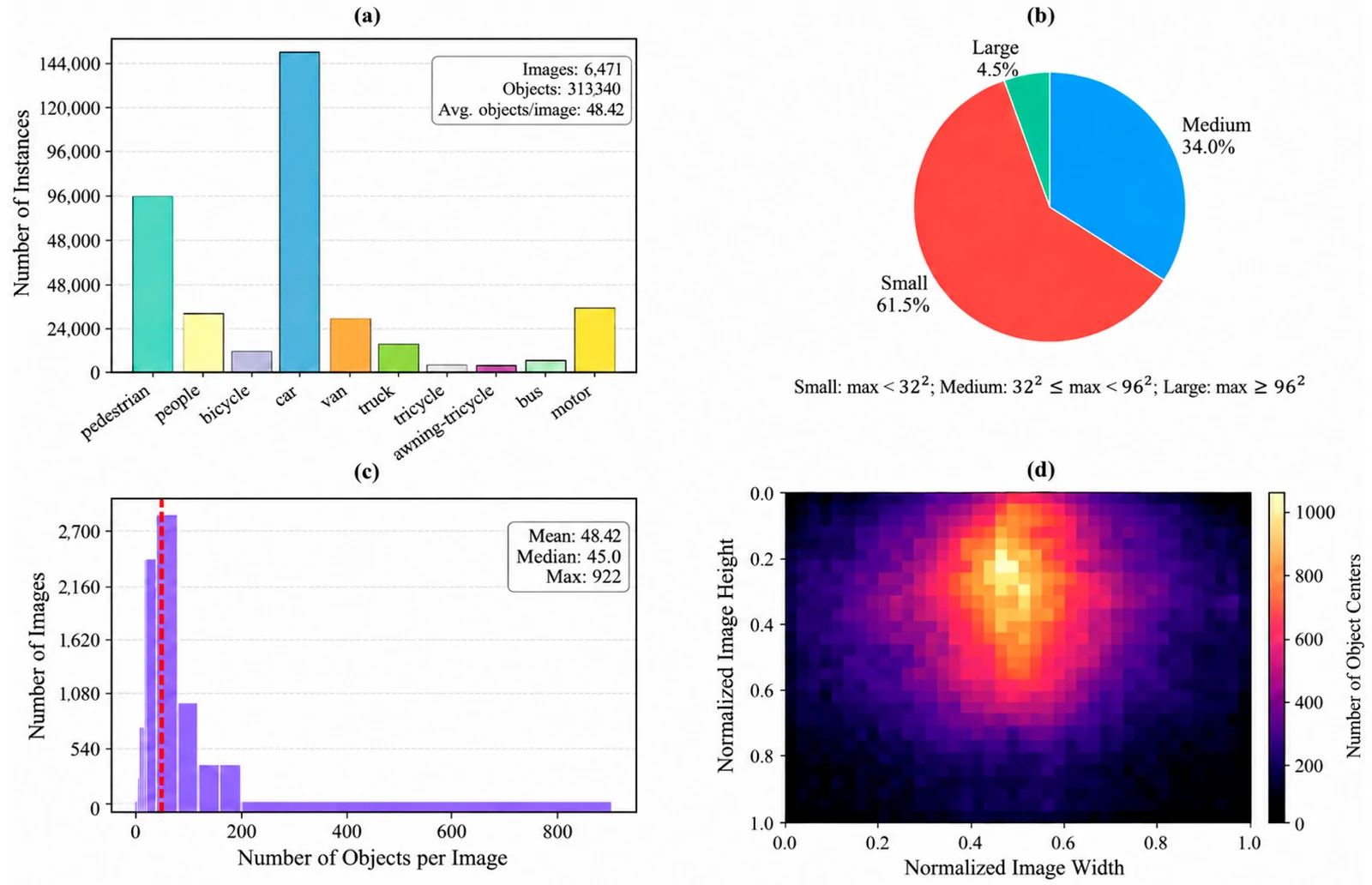
Statistical analysis of detection difficulty in the VisDrone2019 training set: (**a**) class-wise instance distribution; (**b**) object-scale distribution under the COCO criteria; (**c**) number of objects per image, red dashed line: mean = 48.42; (**d**) spatial density of object centers.

**Figure 6:**
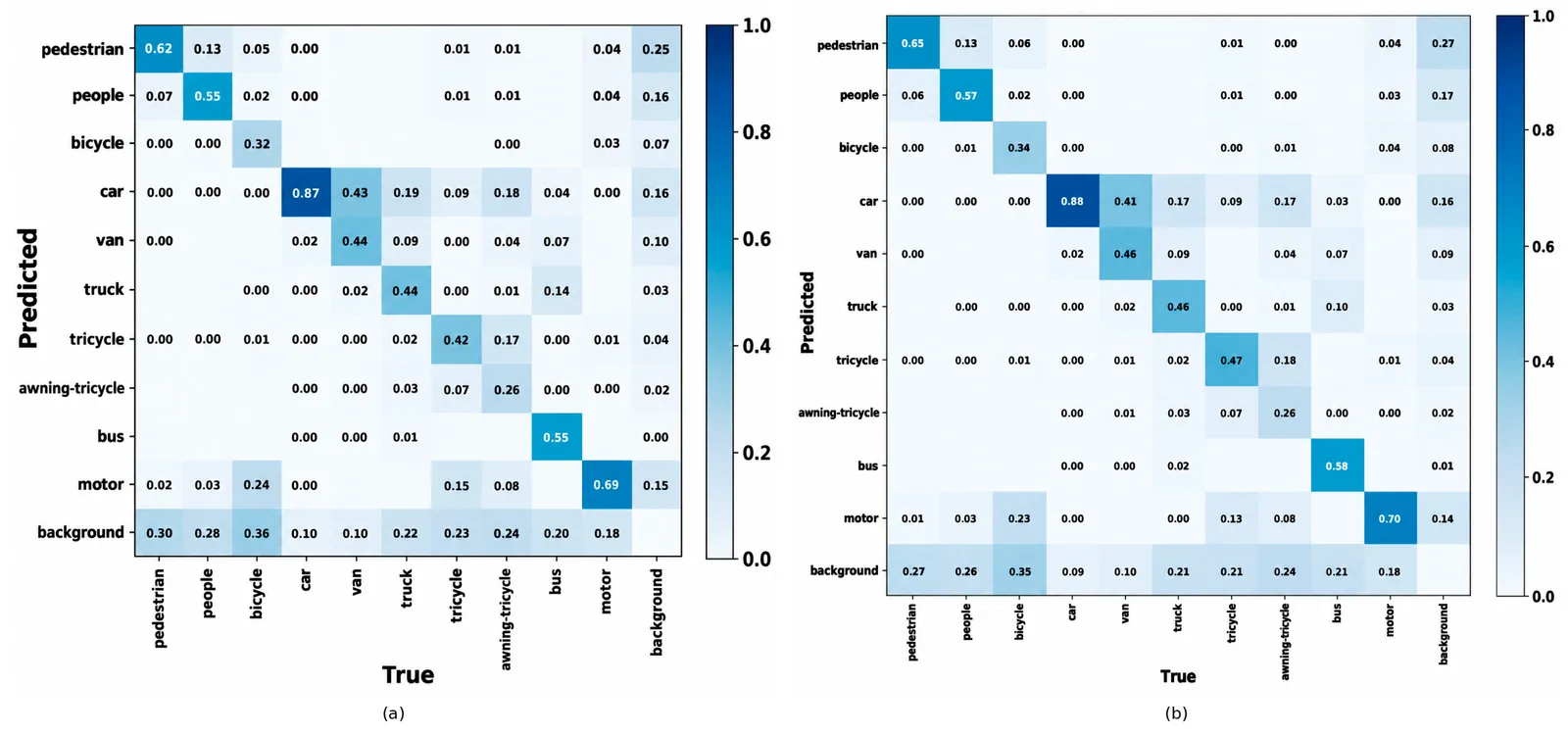
Normalized confusion matrices of RT-DETRv2-R18 and TQ-DETR on VisDrone2019-Val: (**a**) RT-DETRv2-R18; (**b**) TQ-DETR.

**Figure 7:**
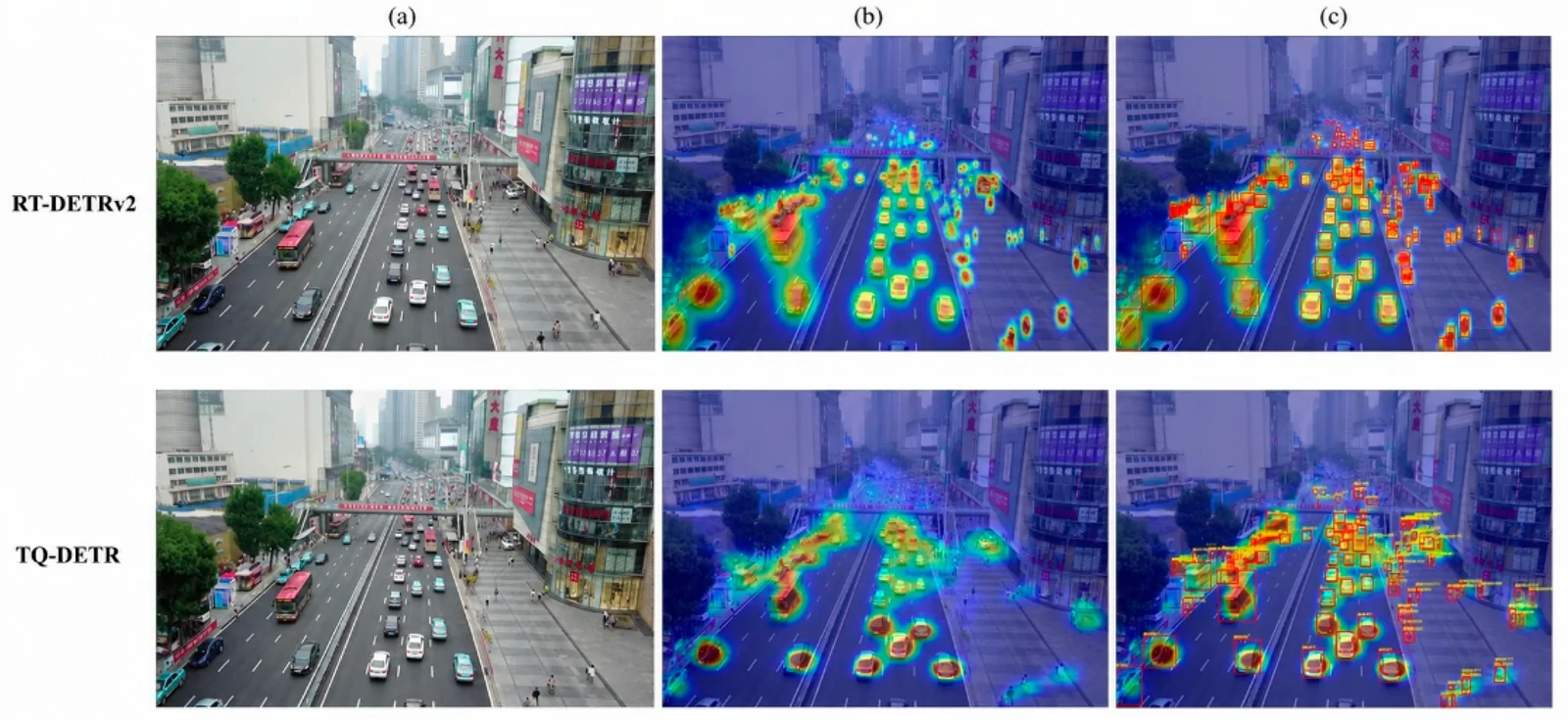
Qualitative comparison in a representative dense UAV scene. (**a**) Original (**b**) Confidence heatmap (**c**) Detection overlay.

**Table 1:** Experimental environment.

Category	Item	Configuration
Hardware	CPU	Intel Xeon Gold 6152 (Intel Corporation, Santa Clara, CA, USA)
	GPU	NVIDIA GeForce RTX 3090 Ti (24 GB; NVIDIA Corporation, Santa Clara, CA, USA)
Software	Operating system	Ubuntu 20.04
	Python	3.8
	CUDA	11.7
	PyTorch	2.0.1

**Table 2:** Core training settings.

Component	Setting
Input size	640 × 640
Batch size	14
Epochs	160
Weight decay	0.0001
Final learning-rate factor	0.1
Initial learning rate	0.0001
Optimizer	AdamW

**Table 3:** Detection performance comparison between RT-DETRv2-R18 and TQ-DETR on the VisDrone2019 validation set, reported as mean ± standard deviation. Bold values indicate improvements over the RT-DETRv2-R18 baseline.

Method	AP	AP_50_	AP_75_	AP_s_	AP_m_	AP_l_
RT-DETRv2 [17]	28.42 ± 0.51	47.53 ± 0.74	28.36 ± 0.62	19.25 ± 0.58	39.67 ± 0.42	58.81 ± 0.54
TQ-DETR (Ours)	**30.21 ± 0.51**	**50.28 ± 0.65**	**30.50 ± 0.61**	**21.06 ± 0.57**	**41.60 ± 0.31**	**62.79 ± 0.30**

**Table 4:** Contextual cross-paper comparison with representative object detectors on the VisDrone2019-DET validation set.

Method	Input Size	AP (%)	AP_50_ (%)	Params (M)	FLOPs (G)
*Two-stage methods*					
Cascade R-CNN [24]	-	14.50	24.70	69.18	-
Faster R-CNN [25]	640 × 640	18.20	31.00	41.20	127.0
*One-stage methods*					
YOLOv12-M [26]	640 × 640	26.30	43.10	20.20	67.50
HIC-YOLOv5 [27]	640 × 640	26.00	44.30	10.50	31.20
MSFE-YOLO-L [28]	640 × 640	29.00	46.80	41.60	160.2
*End-to-end methods*					
DETR [11]	1333 × 750	24.10	40.10	40.00	187.10
Deformable DETR [12]	1333 × 800	27.10	42.20	40.0	173.0
RT-DETRv2-R18	640 × 640	28.42	47.53	19.88	61.19
RT-DETR-R50 [16]	640 × 640	30.90	50.70	41.80	133.2
UAV-DETR-R50 [29]	640 × 640	31.50	51.10	42.00	170.0
VRF-DETR [30]	640 × 640	31.80	51.40	13.50	44.30
TQ-DETR	640 × 640	30.21	50.28	20.51	69.38

**Table 5:** Ablation results for different module combinations. √: included; ×: not included. Bold values indicate the best results.

Method	TOB	DQ	OG-MBF	AP	AP_50_	AP_75_	AP_s_	AP_m_	AP_l_
Baseline model	×	×	×	27.84	46.68	27.66	18.58	39.19	59.01
TOB	√	×	×	27.80	46.83	27.77	18.75	38.93	57.99
TOB + DQ	√	√	×	28.37	47.75	28.18	19.45	39.39	59.00
TOB + OG-MBF	√	×	√	29.29	49.21	29.16	**20.34**	40.52	57.66
TQ-DETR (Ours)	√	√	√	**29.53**	**49.49**	**29.46**	20.30	**40.95**	**62.93**

**Table 6:** Query number analysis.

Query	AP	AP_50_	AP_75_	AP_s_	AP_m_	AP_l_
250 + 150	28.78	48.42	28.53	19.70	39.80	59.54
300 + 100	29.53	49.49	29.46	20.30	40.95	62.93
350 + 50	28.88	48.60	28.84	19.94	40.07	60.20

**Table 7:** Gaussian σ analysis.

Gaussian σ	AP	AP_50_	AP_75_	AP_s_	AP_m_	AP_l_
1.0	28.82	48.47	28.96	19.55	40.12	57.20
2.0	29.53	49.49	29.46	20.30	40.95	62.93
3.0	28.97	48.57	28.83	19.72	40.04	60.00

**Table 8:** Model complexity and detection performance.

Method	Params (M)	FLOPs (G)	FPS	AP (%)	AP_50_ (%)	AP_s_ (%)
RT-DETRv2 [17]	19.88	61.19	81.39	28.42	47.53	19.25
TQ-DETR	20.51	69.38	52.88	30.21	50.28	21.06

**Table 9:** Class-wise ${\mathrm{AP}}_{50}$ on VisDrone2019-Val.

Method	Pedestrian	People	Bicycle	Car	Van	Truck	Tricycle	Awning-Tricycle	Bus	Motor
RT-DETRv2-R18 [17]	52.6	47.0	23.2	83.8	49.2	39.4	34.9	18.6	61.7	56.5
TQ-DETR	56.6	49.9	26.6	85.2	51.2	43.6	37.7	21.3	62.8	59.8

## Data Availability

The VisDrone2019 dataset analyzed in this study is publicly available at https://github.com/VisDrone/VisDrone-Dataset (accessed on 20 September 2026). No new dataset was created in this study. The source code implementing TQ-DETR and additional materials supporting the reported results are available from the corresponding author upon reasonable request.

## References

[1] Zhu P., Wen L., Du D., Bian X., Fan H., Hu Q., Ling H. (2022). Detection and tracking meet drones challenge. IEEE Trans. Pattern Anal. Mach. Intell..

[2] Yang F., Fan H., Chu P., Blasch E., Ling H. Clustered object detection in aerial images. Proceedings of the IEEE/CVF International Conference on Computer Vision (ICCV).

[3] Liu K., Fu Z., Jin S., Chen Z., Zhou F., Jiang R., Chen Y., Ye J. (2025). ESOD: Efficient small object detection on high-resolution images. IEEE Trans. Image Process..

[4] Yang C., Huang Z., Wang N. QueryDet: Cascaded sparse query for accelerating high-resolution small object detection. Proceedings of the IEEE/CVF Conference on Computer Vision and Pattern Recognition (CVPR).

[5] Xu C., Wang J., Yang W., Yu H., Yu L., Xia G.-S. (2022). Detecting tiny objects in aerial images: A normalized Wasserstein distance and a new benchmark. ISPRS J. Photogramm. Remote Sens..

[6] Lin T.-Y., Dollár P., Girshick R., He K., Hariharan B., Belongie S. Feature pyramid networks for object detection. Proceedings of the IEEE Conference on Computer Vision and Pattern Recognition (CVPR).

[7] Deng C., Wang M., Liu L., Liu Y., Jiang Y. (2022). Extended feature pyramid network for small object detection. IEEE Trans. Multimed..

[8] Zhu X., Lyu S., Wang X., Zhao Q. TPH-YOLOv5: Improved YOLOv5 based on Transformer prediction head for object detection on drone-captured scenarios. Proceedings of the IEEE/CVF International Conference on Computer Vision Workshops (ICCVW).

[9] Zhang Z. (2023). Drone-YOLO: An efficient neural network method for target detection in drone images. Drones.

[10] Li Y., Fan Q., Huang H., Han Z., Gu Q. (2023). A modified YOLOv8 detection network for UAV aerial image recognition. Drones.

[11] Carion N., Massa F., Synnaeve G., Usunier N., Kirillov A., Zagoruyko S., Vedaldi A., Bischof H., Brox T., Frahm J.-M. (2020). End-to-end object detection with Transformers. Computer Vision—ECCV 2020.

[12] Zhu X., Su W., Lu L., Li B., Wang X., Dai J. Deformable DETR: Deformable Transformers for end-to-end object detection. Proceedings of the International Conference on Learning Representations (ICLR).

[13] Liu S., Li F., Zhang H., Yang X., Qi X., Su H., Zhu J., Zhang L. DAB-DETR: Dynamic anchor boxes are better queries for DETR. Proceedings of the International Conference on Learning Representations (ICLR).

[14] Zhang H., Li F., Liu S., Zhang L., Su H., Zhu J., Ni L.M., Shum H.-Y. DINO: DETR with improved denoising anchor boxes for end-to-end object detection. Proceedings of the International Conference on Learning Representations (ICLR).

[15] Yao Z., Ai J., Li B., Zhang C. (2021). Efficient DETR: Improving end-to-end object detector with dense prior. arXiv.

[16] Zhao Y., Lv W., Xu S., Wei J., Wang G., Dang Q., Liu Y., Chen J. DETRs beat YOLOs on real-time object detection. Proceedings of the IEEE/CVF Conference on Computer Vision and Pattern Recognition (CVPR).

[17] Lv W., Zhao Y., Chang Q., Huang K., Wang G., Liu Y. (2024). RT-DETRv2: Improved baseline with bag-of-freebies for real-time detection Transformer. arXiv.

[18] Kong Y., Shang X., Jia S. (2024). Drone-DETR: Efficient small object detection for remote sensing image using enhanced RT-DETR model. Sensors.

[19] Liu X., Zou H., Li J., Pan L., He S., Cao X., Zhang Y., Li J., Chen W. (2026). D^2^-DETR: DETR with dual-domain frequency–spatial modeling for unmanned aerial vehicle imagery object detection. Knowl.-Based Syst..

[20] Wang Z., Yu P., Zhang X., Sun H. (2025). BAP-DETR: Efficient drone object detection network based on bipartite attentive processing and dual fusion encoder. Comput. Vis. Image Underst..

[21] Liu Y., He M., Hui B. (2025). ESO-DETR: An Improved Real-Time Detection Transformer Model for Enhanced Small Object Detection in UAV Imagery. Drones.

[22] Szegedy C., Ioffe S., Vanhoucke V., Alemi A.A. Inception-v4, Inception-ResNet and the impact of residual connections on learning. Proceedings of the Thirty-First AAAI Conference on Artificial Intelligence.

[23] Touvron H., Cord M., Sablayrolles A., Synnaeve G., Jégou H. Going deeper with image Transformers. Proceedings of the IEEE/CVF International Conference on Computer Vision.

[24] Cai Z., Vasconcelos N. Cascade R-CNN: Delving into high quality object detection. Proceedings of the IEEE/CVF Conference on Computer Vision and Pattern Recognition (CVPR).

[25] Ren S., He K., Girshick R., Sun J. (2015). Faster R-CNN: Towards real-time object detection with region proposal networks. Adv. Neural Inf. Process. Syst..

[26] Tian Y., Ye Q., Doermann D. (2025). YOLOv12: Attention-centric real-time object detectors. Adv. Neural Inf. Process. Syst..

[27] Tang S., Zhang S., Fang Y. HIC-YOLOv5: Improved YOLOv5 for small object detection. Proceedings of the 2024 IEEE International Conference on Robotics and Automation (ICRA).

[28] Qi S., Song X., Shang T., Hu X., Han K. (2024). MSFE-YOLO: An improved YOLOv8 network for object detection on drone view. IEEE Geosci. Remote Sens. Lett..

[29] Zhang H., Zhang H., Liu K., Gan Z., Zhu G.-N. UAV-DETR: Efficient end-to-end object detection for unmanned aerial vehicle imagery. Proceedings of the 2025 IEEE/RSJ International Conference on Intelligent Robots and Systems (IROS).

[30] Liu W., Shi L., An G. (2025). An efficient aerial image detection with variable receptive fields. Remote Sens..

